# Desiccation tolerance in *Acinetobacter baumannii* is mediated by the two-component response regulator BfmR

**DOI:** 10.1371/journal.pone.0205638

**Published:** 2018-10-11

**Authors:** John M. Farrow, Greg Wells, Everett C. Pesci

**Affiliations:** Department of Microbiology and Immunology, The Brody School of Medicine at East Carolina University, Greenville, North Carolina, United States of America; Centre National de la Recherche Scientifique, Aix-Marseille Université, FRANCE

## Abstract

For the opportunistic pathogen *Acinetobacter baumannii*, desiccation tolerance is thought to contribute significantly to the persistence of these bacteria in the healthcare environment. We investigated the ability of *A*. *baumannii* to survive rapid drying, and found that some strains exhibited a profoundly desiccation-resistant phenotype, characterized by the ability of a large proportion of cells to survive on a dry surface for an extended period of time. However, this phenotype was only displayed during the stationary phase of growth. Most interestingly, we found that drying resistance could be lost after extended cultivation in liquid medium. Genome sequencing of isolates that became drying-sensitive identified mutations in *bfmR*, which encodes a two-component response regulator that is important for *A*. *baumannii* virulence. Additionally, BfmR was necessary for the expression of stress-related proteins during stationary phase, and one of these, KatE, was important for long-term drying survival. These results suggested that BfmR may control stress responses, and we demonstrated that the Δ*bfmR* mutant was more sensitive to hydrogen peroxide, nutrient starvation, and increased osmolarity. We also found that cross-protection against drying could be stimulated by either starvation, which required BfmR, or increased osmolarity. These results imply that BfmR plays a role in controlling stress responses in *A*. *baumannii* which help protect cells during desiccation, and they provide a regulatory link between this organism’s ability to persist in the environment and pathogenicity.

## Introduction

The gram-negative bacterium *Acinetobacter baumannii* is an opportunistic pathogen that primarily infects severely ill or wounded individuals, but it rarely causes disease outside of the healthcare setting [1]. These infections have become a growing concern in recent years due to the ability of this organism to develop multi-drug resistance [2]. In contrast to many other *Acinetobacter* species, which are mostly non-pathogenic and can be isolated from a wide variety of environmental sources, *A*. *baumannii* is most frequently found in association with hospitalized patients or in the nosocomial environment, where it can become a persistent contaminant [3, 4]. Sustained colonization of the hospital environment by *A*. *baumannii* can lead to outbreaks of infection, particularly in intensive care units and trauma centers [5–8]. This was clearly illustrated during U.S. military operations in Iraq and Afghanistan during 2001–2007, where more than 700 wounded soldiers were either infected or colonized with *A*. *baumannii* during passage through the military evacuation chain [9, 10]. One trait that is thought to aid in the survival of *A*. *baumannii* in the clinical environment is its ability to tolerate desiccation.

Desiccation is a common environmental stress that poses many challenges to bacterial cells. Water loss leads to decreased turgor pressure and biochemical changes that can damage cell membranes, and drying can also cause denaturation of intracellular proteins and conformational changes to DNA [11, 12]. Additionally, reactive oxygen species generated during desiccation can damage both proteins and DNA [11, 13]. In response, bacteria have developed a variety of mechanisms to mitigate the damage from drying. Some species can differentiate into dormant forms such as endospores or cysts, which are extremely desiccation-resistant, but this strategy requires a significant expenditure of time and resources [14, 15]. Other species have developed mechanisms that either help protect susceptible cellular components from damage, or that sequester water in an attempt to avoid dehydration. These mechanisms include the alteration of membrane composition or LPS modification to help stabilize membranes during drying, and the accumulation of compatible solutes such as trehalose, which can protect cytoplasmic and membrane constituents [11, 14]. Alternatively, exopolysaccharide production and biofilm formation can be a way to retain water in the local microenvironment and protect bacterial cells during dry conditions [16]. Yet while many bacterial species possess these capabilities, their ability to tolerate desiccation can vary widely. Drying survival times can range from minutes to years, and there can even be considerable variation between different strains of the same species [11]. Therefore, the molecular, biophysical, and regulatory processes that determine desiccation tolerance in non-spore forming bacteria remains a poorly understood aspect of bacterial physiology.

Compared to *Escherichia coli* and *Pseudomonas aeruginosa*, two other Gram-negative pathogens commonly found in the nosocomial environment, *A*. *baumannii* typically displays a greater ability to survive drying in laboratory tests, but the survival of different *A*. *baumannii* strains when dried can be variable, ranging from a few days to months [17–19]. Overall, laboratory studies have shown that many clinical *A*. *baumannii* strains can survive at least 20 days on dry surfaces, and some can survive 100 days or more [20–22]. *A*. *baumannii* has also been isolated from many different sources in the hospital environment, such as bed rails, pillows, mattresses, tables, and medical equipment [8]. These findings indicate that transfer of this pathogen via fomites is a major issue in clinical environments, and is most likely sustained by this organism’s ability to withstand desiccation and prolonged dry conditions. While this points to the importance of desiccation tolerance for this opportunistic pathogen, only a few specific factors that contribute to this property have been identified. A recent study showed that hepta-acylated lipid A, a component of the *A*. *baumannii* outer membrane, is important for drying survival [23]. Another study found that RecA, an important cellular factor for DNA repair, was required for cells to withstand desiccation [24]. Additional investigations have also suggested that biofilm formation or transition to a dormant state could be important for desiccation survival [25, 26]. While these studies have provided valuable insights, they do not provide a comprehensive understanding of *A*. *baumannii* desiccation tolerance.

We decided to further investigate this property of *A*. *baumannii* since it likely helps this opportunistic pathogen to come in contact with hospitalized individuals that are most susceptible to infection. We assessed the drying survival of a small group of *A*. *baumannii* strains, and confirmed that different strains appear to have distinct desiccation tolerance phenotypes. We also discovered that drying-tolerant strains can convert to a drying-sensitive phenotype during laboratory cultivation, and analysis of these strains led us to establish a role for the regulator BfmR in desiccation tolerance and other stress-related phenotypes.

## Results

### Some *A*. *baumannii* strains display an extremely desiccation-tolerant phenotype

Previous studies found that *A*. *baumannii* strains can vary widely in their ability to survive desiccation, with some only surviving a few days, and others being able to survive for weeks or months in a dried state [17]. To identify suitable strains for studying desiccation tolerance we tested the ability of a small group of *A*. *baumannii* strains to withstand drying. For these experiments we harvested cells from stationary phase broth cultures, washed the cells with water, and allowed small samples of washed cells to dry on a plastic surface (see Materials and methods). Bacterial samples were visibly dry in 30 min to 1 h under the conditions tested. This procedure allowed us to focus on the intrinsic properties of cells that promote desiccation tolerance, since media components can affect bacterial drying survival [17, 18]. Additionally, we believed that this method would reasonably simulate a bacterial contamination event that could occur in the nosocomial environment. We began by examining the maximum survival time of dried *A*. *baumannii* cells after incubation at ambient temperature and humidity (19–23°C, 25–61% RH). The results from these experiments showed that this group of *A*. *baumannii* strains displayed three different desiccation-tolerance phenotypes (Table 1). One widely studied strain, ATCC 19606, had low desiccation tolerance under these conditions, losing viability after a few days of drying. A second group of strains, including another commonly studied strain, ATCC 17978, had moderate resistance to drying, surviving for an average of 20–43 days. The maximum survival times for this group of strains were quite variable, and were likely influenced by fluctuations in relative humidity [17], which ranged from 25–61% based on regular monitoring of the ambient conditions. However, another set of strains consistently survived desiccation for 90 days or longer. We defined these strains as having a profoundly desiccation-tolerant phenotype.

**Table 1:** Maximum survival time of dried *A*. *baumannii* cells.

Strain	Average survival time in days (mean ± SD)^1^	Average percent survival after 90 days
ATCC 19606	3 ± 1	0
A118	20 ± 7	0
ATCC 17978	34 ± 22	0
ATCC 17904	22 ± 12	0
AB09-002	43 ± 26	0
AB09-003	> 90	5%
ATCC 17961	> 90	6%
AB5075	~ 90†	< 0.01%

^1^ Washed *A*. *baumannii* cells were spotted on a smooth polystyrene surface and allowed to air dry. Dried cells were incubated at 19–23°C (mean 22°C), with a relative humidity of 25–61% (mean 46%). Results are from at least three separate experiments.

^†^ Average survival was approximately 90 days, based on the following results from four independent experiments: > 90 days, > 90 days, 44 days, 90 days.

**Fig 1:**
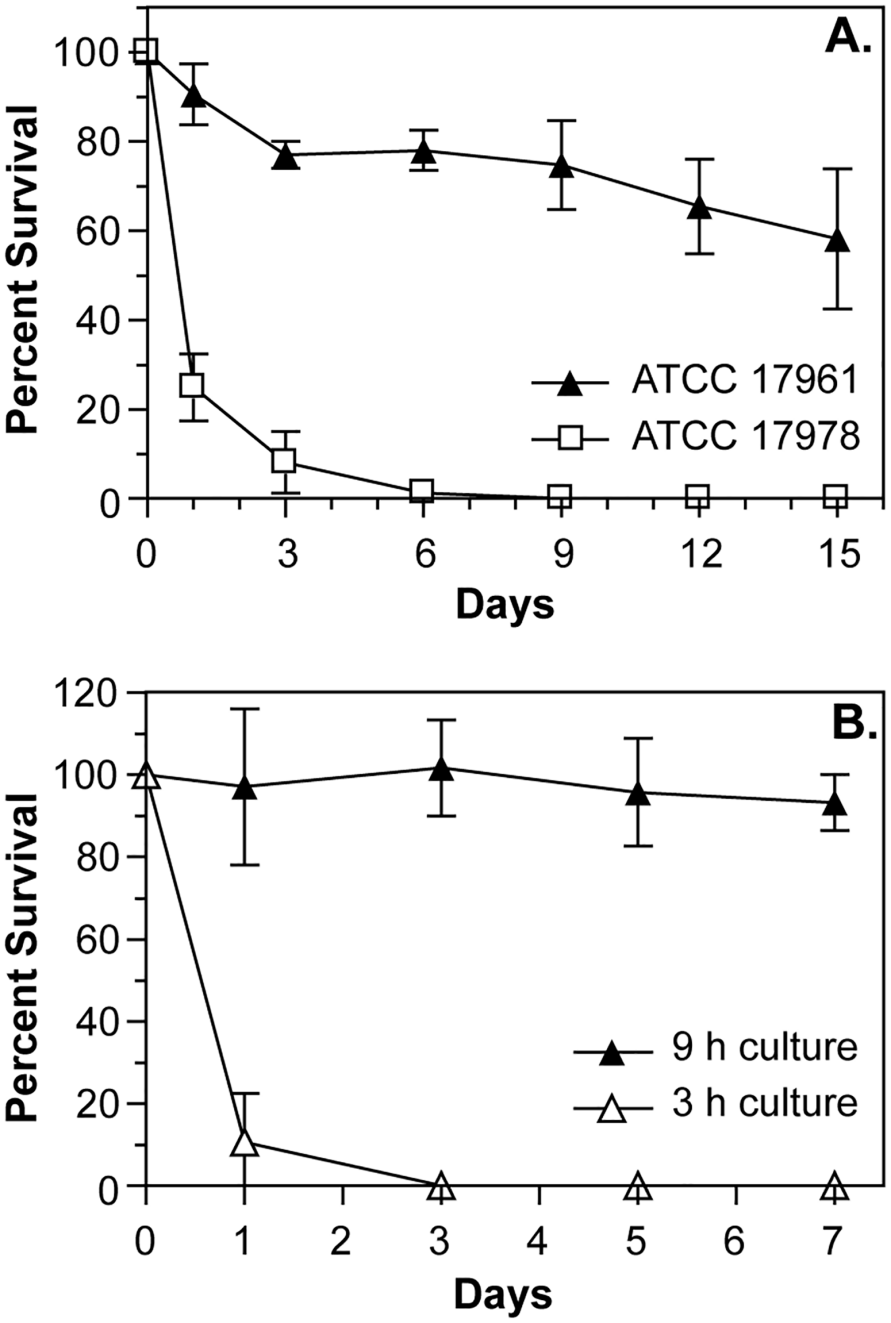
Desiccation survival phenotypes of *A*. *baumannii* cells. (A) *A*. *baumannii* strains were grown overnight in LB medium, and then cells were washed with water and samples were dried on polystyrene. Dried cells were incubated at 25°C and 80% RH, and at the indicated times dried samples were suspended, and survival was assessed by CFU counts. (B) Cells from cultures of *A*. *baumannii* strain ATCC 17961 were harvested after either 3 h (logarithmic phase) or 9 h (stationary phase) of growth in LB medium, washed with water, dried, and then survival was assessed at the indicated times. The data presented are the mean ± SD from at least three independent experiments.

The difference between moderately and profoundly desiccation-tolerant strains was even more apparent when we examined the survival curves of *A*. *baumannii* cells during the first two weeks of drying. We performed these and all following experiments under conditions of controlled temperature and humidity (24–26°C and 79–81% RH) to minimize variations in survival caused by these factors. Moderately drying-resistant strains, represented by strain ATCC 17978, had a large decrease in the number of viable cells during the first week of desiccation, with only a small proportion (less than 1%) of the population being able to survive being dried for an extended period of time (Fig 1A). We observed similar patterns of survival for other moderately desiccation-tolerant strains A118, ATCC 17904, and AB09-002 (S1 Fig). In contrast, for profoundly desiccation-tolerant strains, represented by strain ATCC 17961, we saw only a slight decrease in the number of viable cells after the first few days of drying, followed by a slow decline in viability (Fig 1A). This survival pattern was also displayed by strains AB09-003 and AB5075 (S1 Fig). In addition, we observed similar trends in drying survival for strains ATCC 17978 and AB09-003 under conditions of lower relative humidity (25% RH, 24–26°C). These results showed that profound desiccation tolerance is a distinct phenotype of certain *A*. *baumannii* strains, characterized by the survival of a large proportion of the cells when dried for an extended period of time.

We also made another interesting observation about the desiccation-tolerant phenotype. In our initial experiments we tested the survival of cells harvested from the stationary phase of growth, but we saw large differences in drying survival when we tested cells taken from the logarithmic growth phase. Compared with cells that were grown in lysogeny broth (LB) for 9 h, cells from strain ATCC 17961 that grew for only 3 h had a greatly reduced ability to survive desiccation, and instead generated a survival curve that was more similar to strains with moderate to low desiccation tolerance (Fig 1B). Cells taken from logarithmic phase cultures of strains AB09-003 and AB5075 also had a greatly decreased ability to survive drying. These results implied that factors which are important for desiccation tolerance in *A*. *baumannii* are regulated in a growth phase-dependent manner.

### Laboratory adaptation reveals a regulator that is required for desiccation tolerance

**Fig 2:**
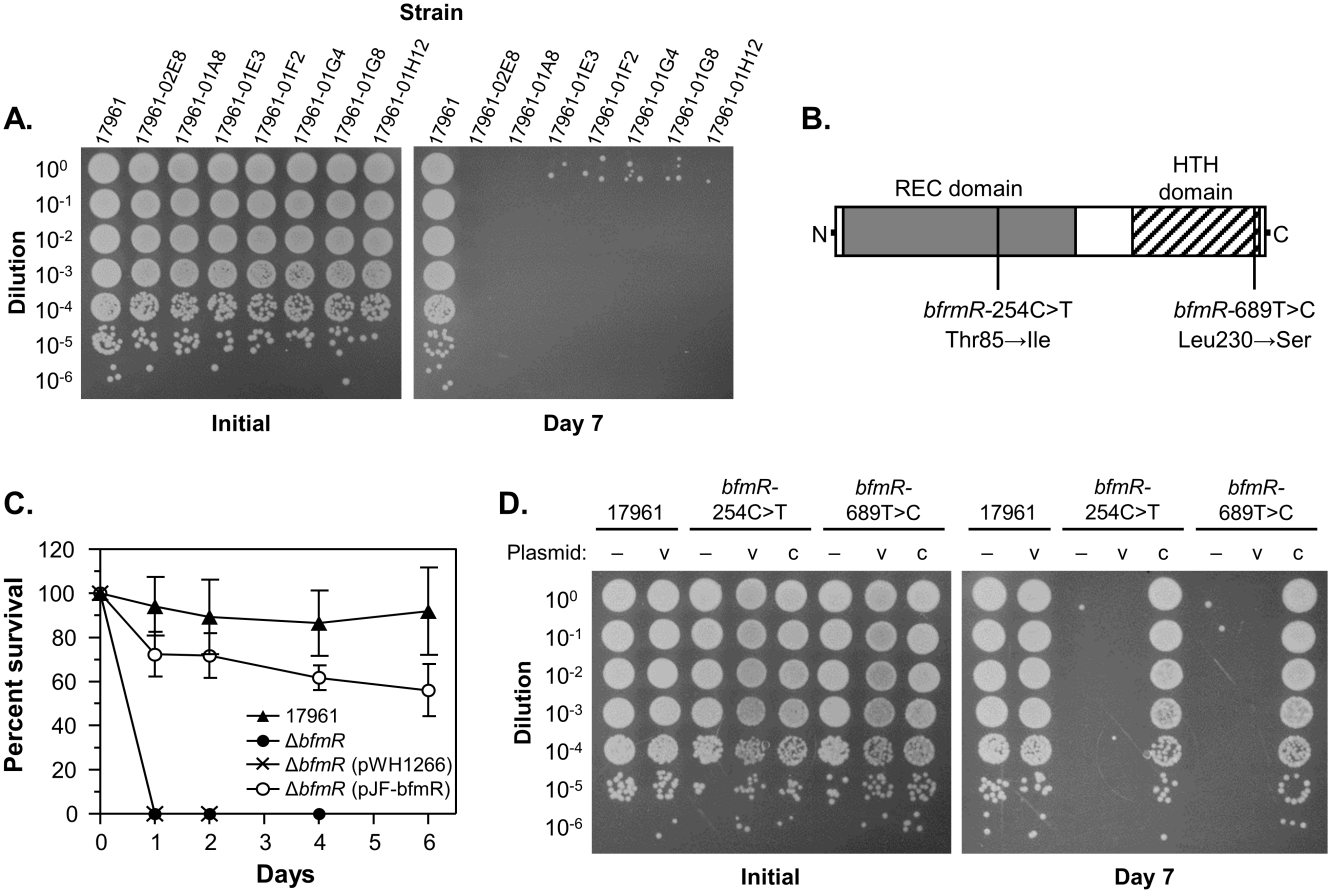
The two-component response regulator BfmR is required for desiccation tolerance. *A*. *baumannii* strains were grown overnight in LB medium, and then cells were washed with water, dried, and assessed for desiccation tolerance at 25°C and 80% RH. (A) The survival of dried cells from the wild-type strain ATCC 17961 and derivatives isolated after extended culture in LB medium were tested. Samples from the initial inoculum and after 7 days of drying were diluted and spotted onto the surface of an LB plate. These data are representative of at least two independent experiments. (B) A representation of the BfmR protein showing the relative positions of the receiver domain (REC domain, gray box), the DNA-binding helix-turn-helix domain (HTH domain, hashed box), and the nucleotide and corresponding amino acid substitutions identified in the strains tested in panel (A). (C) *A*. *baumannii* strains ATCC 17961 (solid triangle), 17961-Δ*bfmR* (solid circle), 17961-Δ*bfmR* carrying vector plasmid pWH1266 (X), and 17961-Δ*bfmR* carrying an intact copy of *bfmR* on plasmid pJF-*bfmR* (open circle) were assessed for drying survival on the indicated days. The data presented are the mean ± SD from at least four independent experiments. (D) Cells from strains ATCC 17961, 17961-*bfmR*-254C>T, and 17961-*bfmR*-689T>C carrying either no plasmid (–), vector plasmid pWH1266 (v), or plasmid pJF-*bfmR* for complementation (c) were tested for survival after drying. Samples from the initial inoculum and after 7 days of drying were diluted and spotted onto the surface of an LB plate. These data are representative of at least two independent experiments.

A previous study that examined the drying survival of a larger group of *A*. *baumannii* strains noted that more recent isolates tended to be more desiccation tolerant than laboratory strains [17]. Since bacteria are rarely, if ever, subjected to dry conditions in the laboratory, we hypothesized that desiccation resistance could be lost during laboratory cultivation, and that this could be a route to understanding the molecular mechanisms that allow *A*. *baumannii* to survive drying. To test this, we cultured the highly desiccation-resistant strain ATCC 17961 for five to seven passes in liquid medium, then isolated individual clones and screened them for drying survival. We then performed a more controlled test for drying survival on a subset of clones that appeared to have reduced desiccation tolerance, and were able to readily identify drying-sensitive isolates derived from each strain (Fig 2A). Unfortunately, our primary screening procedure produced a large number of false-positive results, so we were unable to accurately determine the frequency at which drying-sensitive clones arose in the population. However, we were able to isolate sensitive clones in experiments where only approximately 200 clones were screened, suggesting that desiccation resistance can be lost at a fairly high rate.

Recently, two different mechanisms have been described to account for frequent phenotypic variations in *A*. *baumannii*. The first involves the loss of a large, self-transmissible plasmid during laboratory cultivation [27]. Strain 17961 did not appear to carry a large pAB3/pAB04-1-type plasmid as assessed by PCR screening, but it does harbor at least two other plasmids. Monitoring of these plasmids did not detect any difference in the number or size of plasmids present in the drying-sensitive derivatives of 17961, which indicates that the observed changes in desiccation tolerance were not due to plasmid loss. The second mechanism involves a cell density-dependent phase change that was first observed in strain AB5075 [28]. We viewed colonies of ATCC 17961 and the drying-sensitive derivatives under the appropriate conditions to observe phase change (growth on 0.5X LB, 0.8% agar), and we did not observe any changes in colony opacity, although we were able to see both opaque and translucent colony variants for our control strain AB5075. Additionally, we took two of our drying-sensitive isolates, cultured them repeatedly in LB medium, and repeated our screening procedure to see if we could detect any reversion to a drying-resistant phenotype. We did not isolate any desiccation-tolerant clones from these experiments. Together, these results strongly suggested that the variation in drying-resistance phenotypes that we observed was not the result of a phase change mechanism.

To identify any genetic changes that could be the cause of the change in desiccation tolerance, we analyzed the genome of strain ATCC 17961 and seven drying-sensitive derivatives of this strain by high-throughput DNA sequencing. First, we generated a draft genome sequence for strain 17961, which we have made available on the PATRIC website (http://www.patricbrc.org). The total length of the generated contigs was 4,057,452 bp, with a 39.2% GC content, which is similar to genomes of other *A*. *baumannii* strains. Next, we aligned sequencing reads from each of the drying-sensitive isolates to the parent genome and identified variations. For six of the isolates (17961-02E8, 17961-01E3, 17961-01F2, 17961-01G4, 17961-01G8, and 17961-01H12) we identified the same two single-nucleotide variations: insertion of a “T” residue in an intergenic region upstream from a gene encoding a predicted fimbrial protein precursor (17961 genome feature 470.2202.peg.2098), and substitution of a “C” residue for a “T” at bp 689 of the *bfmR* gene, leading to the conversion of BfmR Leu230 to Ser. These six isolates were collected during the same experiment, and likely represent the expansion of a single clone. In the seventh isolate (17961-01A8) we identified a single nucleotide substitution at bp 254 of *bfmR* (C to T), converting BfmR Thr85 to Ile. Since these analyses identified two independent, nonsynonymous mutations in *bfmR*, we decided to further investigate the role of *bfmR* in desiccation tolerance.

The *bfmR* gene encodes an OmpR/PhoB-family bacterial two-component response regulator. Like other members of this family, BfmR contains an N-terminal receiver domain, which can be phosphorylated at a conserved aspartate residue to control protein function, and a C-terminal helix-turn-helix DNA binding domain (Fig 2B). Phosphorylation of BfmR is likely controlled by the sensor histidine kinase BfmS, which is co-transcribed with *bfmR* [29], and which controls the expression of many of the same genes as BfmR [30]. Since it was unclear how each independent point mutation that we identified would affect the function of BfmR, we constructed an isogenic, in-frame *bfmR* deletion mutant in strain 17961. We also re-introduced the *bfmR*-254C>T and *bfmR*-689T>C point mutations onto the strain 17961 chromosome by allelic exchange. We then tested each of these newly constructed strains for the ability to survive desiccation. Compared to the wild-type strain, the *bfmR* mutant had a greatly decreased ability to survive drying, and we were unable to recover any viable cells from the Δ*bfmR* strain after more than four days of drying (Fig 2C). Desiccation tolerance could be restored to the *bfmR* deletion mutant by supplying an intact copy of *bfmR* on a multi-copy plasmid, but was not restored by the vector plasmid alone (Fig 2C). Similarly, each of the strains with point mutations in *bfmR* had a large decrease in survival after drying, and survival was restored by supplying a wild-type copy of *bfmR* to these strains (Fig 2D). These results implied that the *bfmR*-254C>T and *bfmR*-689T>C mutations inactivated BfmR, and indicated that BfmR is required for desiccation tolerance in strain 17961.

### BfmR controls the expression of stress-related proteins during the stationary phase of growth

**Fig 3:**
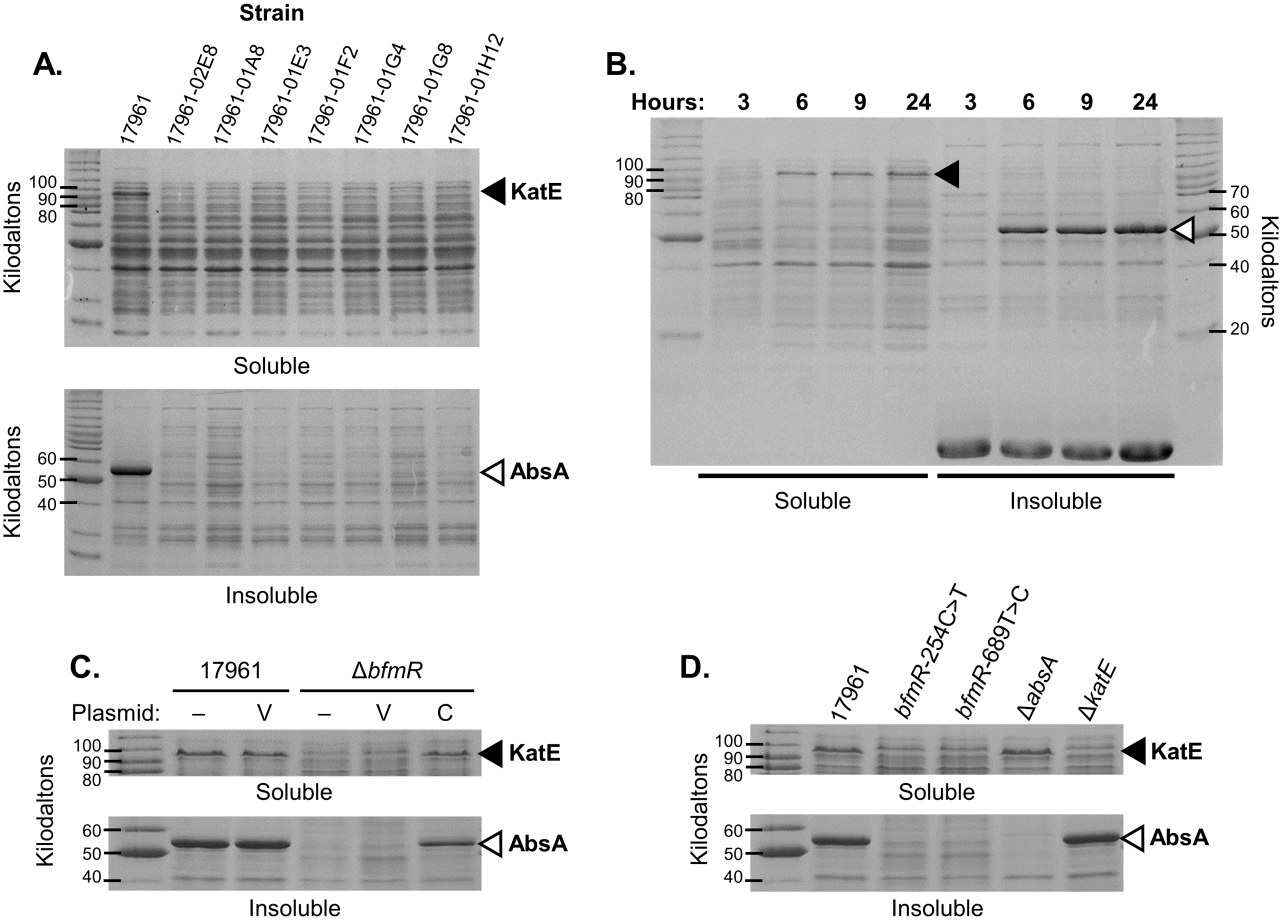
Identification of proteins that are differentially expressed in desiccation tolerant versus sensitive *A*. *baumannii* cells. Lysates from *A*. *baumannii* cells were separated into soluble and insoluble fractions. Samples from each strain containing approximately equivalent amounts of total protein were analyzed by SDS-PAGE, and gels were stained with Coomasie-R250. The positions of KatE and AbsA are indicated by closed and open arrowheads, respectively. These data are representative of at least two independent experiments. (A) Samples from the wild-type strain ATCC 17961, and derivatives of strain 17961 that were isolated after extended culture in LB medium, were analyzed for protein expression. (B) Cells from strain ATCC 17961 were harvested after 3, 6, 9, and 24 h of growth in LB medium and analyzed. (C) Protein expression was analyzed in cells from strains ATCC 17961 and 17961-Δ*bfmR* carrying either no plasmid (–), vector plasmid pWH1266 (v), or plasmid pJF-*bfmR* for complementation (c). (D) Protein expression was examined in strains ATCC 17961, 17961-*bfmR*-254C>T, 17961-*bfmR*-689T>C, 17961-Δ*absA*, and 17961-Δ*katE*.

While analyzing the genome sequences of strain 17961 and drying-sensitive derivatives of this strain, we decided to also examine protein expression profiles to try to identify differences that could be important for desiccation tolerance. Cells for each strain were disrupted using a commercial lysis reagent (BugBuster protein extraction reagent, EMD, Millipore), and then we analyzed the soluble and insoluble fractions of each lysate by SDS-PAGE. Interestingly, we saw that the production of two different proteins appeared to be greatly reduced in the drying-sensitive isolates (Fig 3A). We additionally observed that the expression of these proteins was increased during the stationary phase of growth in strains 17961 (Fig 3B) and AB09-003 (data not shown). We excised the bands corresponding to each of these proteins from polyacrylamide gels and identified them by mass spectrometry. The protein present in the soluble fraction of lysates was identified as the KatE catalase, which aids in the detoxification of hydrogen peroxide. Consistent with our observations, another study showed that *katE* expression was greatly increased during the stationary phase of growth in *A*. *baumannii* [31]. The protein present in the insoluble fraction of lysates is an acidophilic repeat motif-containing protein that appears to be encoded in the genome of many *A*. *baumannii* strains, as assessed by BLAST (http://www.ncbi.nlm.nih.gov/blast). This protein is annotated as a stress-induced protein in some *A*. *baumannii* genomes due to the presence of a conserved N-terminal domain that is present in a variety of stress-related proteins in other bacterial species. Therefore we designated this protein as *A*. *baumannii* stress-related protein A (AbsA).

**Fig 4:**
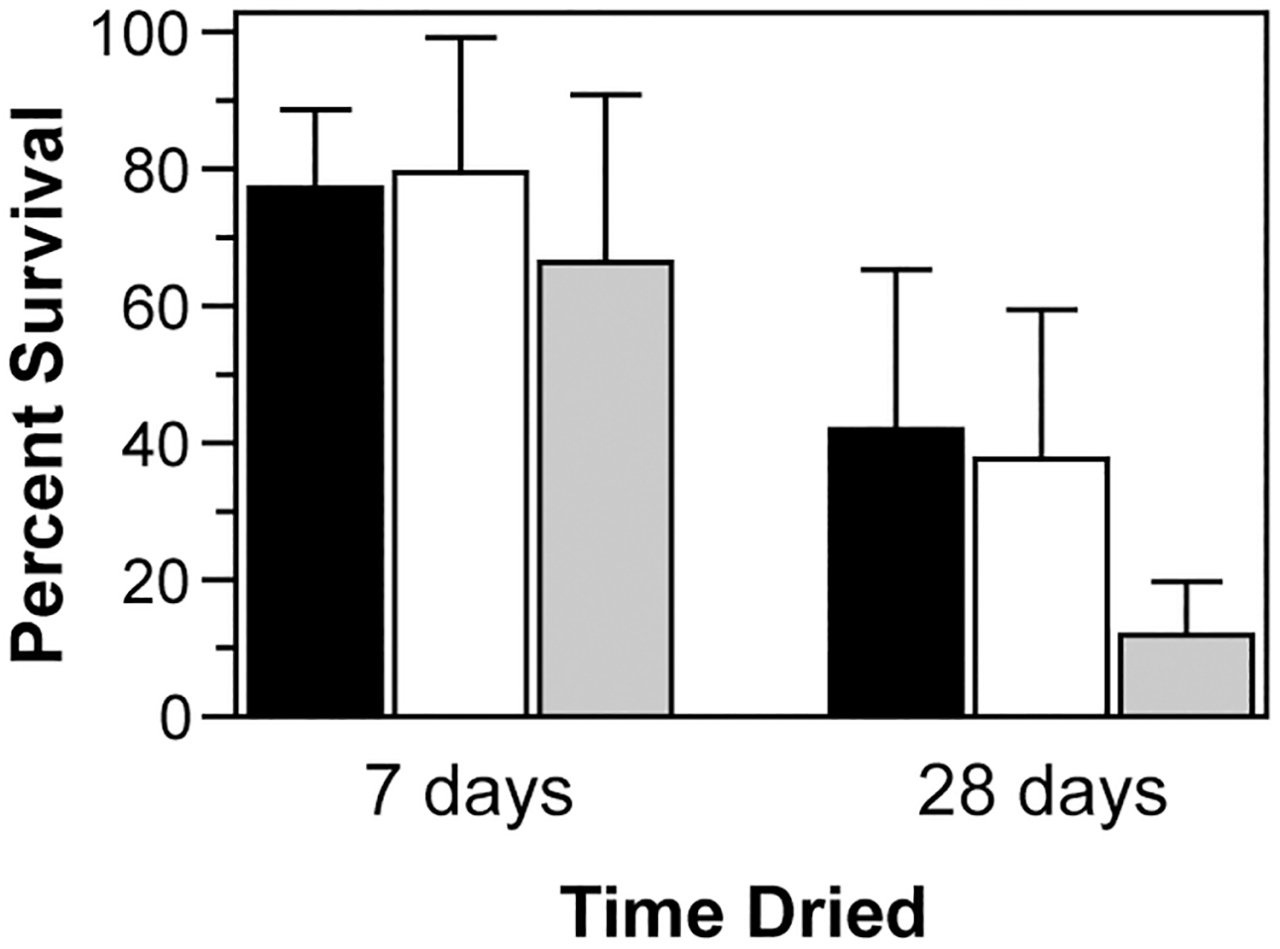
Effects of KatE and AbsA on desiccation tolerance. Dried cells from the wild-type strain ATCC 17961 (black bars), the *absA* deletion mutant 17961-Δ*absA* (white bars), or the *katE* deletion mutant 17961-Δ*katE* (gray bars) were assessed for survival after 7 and 28 days of desiccation at 25°C and 80% RH. [Note: For comparison, the *bfmR* deletion mutant had <1% survival after one day of drying (Fig 2C)]. Data represent the mean ± SD from at least five independent experiments.

Since BfmR is expected to be a regulator of gene expression, we wondered if the decreased levels of KatE and AbsA observed in the drying-sensitive isolates from strain 17961 was a consequence of *bfmR* inactivation. To examine this possibility we analyzed cell lysates from the *bfmR* deletion mutant and the *bfmR*-254C>T and *bfmR*-689T>C mutant strains. We saw that KatE levels appeared to be lower in these strains, and AbsA levels appeared to be greatly reduced (Fig 3C and 3D). KatE and AbsA expression could be restored to the Δ*bfmR* strain by supplying an intact copy of *bfmR* on a plasmid, but not by the vector plasmid alone (Fig 3C). These results indicated that BfmR positively regulates the production of both KatE and AbsA. To test if the loss of either KatE or AbsA could explain the large decrease in desiccation tolerance displayed by the *bfmR* mutant, we constructed strains with in-frame deletions in either *katE* or *absA*. We observed that KatE appeared to be expressed in the Δ*absA* mutant at a level similar to the wild-type, and that AbsA was produced at a wild-type level in the Δ*katE* mutant (Fig 3D), implying that *bfmR* was not inactivated in these strains during laboratory manipulations, and that deletion of either of these genes did not affect expression of the other. Next, we tested the ability of these strains to survive desiccation. We saw that the Δ*katE* and Δ*absA* mutant strains survived just as well as the wild-type strain after seven days of drying (Fig 4). After 28 days of drying the Δ*absA* mutant still survived as well as the wild-type, but there was a significant decrease in the viability of the Δ*katE* mutant strain compared to strain 17961 (Fig 4, Δ*katE* vs. 17961 at 28 days, *P* = 0.02 as determined by *t*-test). However, these results were not sufficient to explain the large decrease (>99%) in viability seen when Δ*bfmR* mutant cells were dried for only a few days (Fig 2C), showing that additional factors that contribute to desiccation tolerance must be absent in the Δ*bfmR* mutant.

### BfmR is important for phenotypes associated with the general stress response

Although we did not identify a specific BfmR-controlled factor that was fully responsible for drying survival, the fact that we found BfmR controls two potentially stress-related proteins that were also upregulated during the stationary phase of growth caused us to consider a role for BfmR as a stress response regulator. In particular, we noted that *katE* expression in *E*. *coli*, despite its role in protecting cells from oxidative stress, is not stimulated by oxidative stress [32]. Instead, *katE* is upregulated during the stationary phase of growth as part of the general stress response [33]. In Gram-negative bacteria the general stress response involves the induction of a large number of genes that provide cross-protection against a variety of environmental stresses [34]. Considering this, we hypothesized that BfmR may coordinate multiple stress responses, allowing *A*. *baumannii* cells to combat the various stresses encountered during desiccation. To examine this, we tested the susceptibility of the wild-type and *bfmR* mutant strains to a variety of stressors.

**Fig 5:**
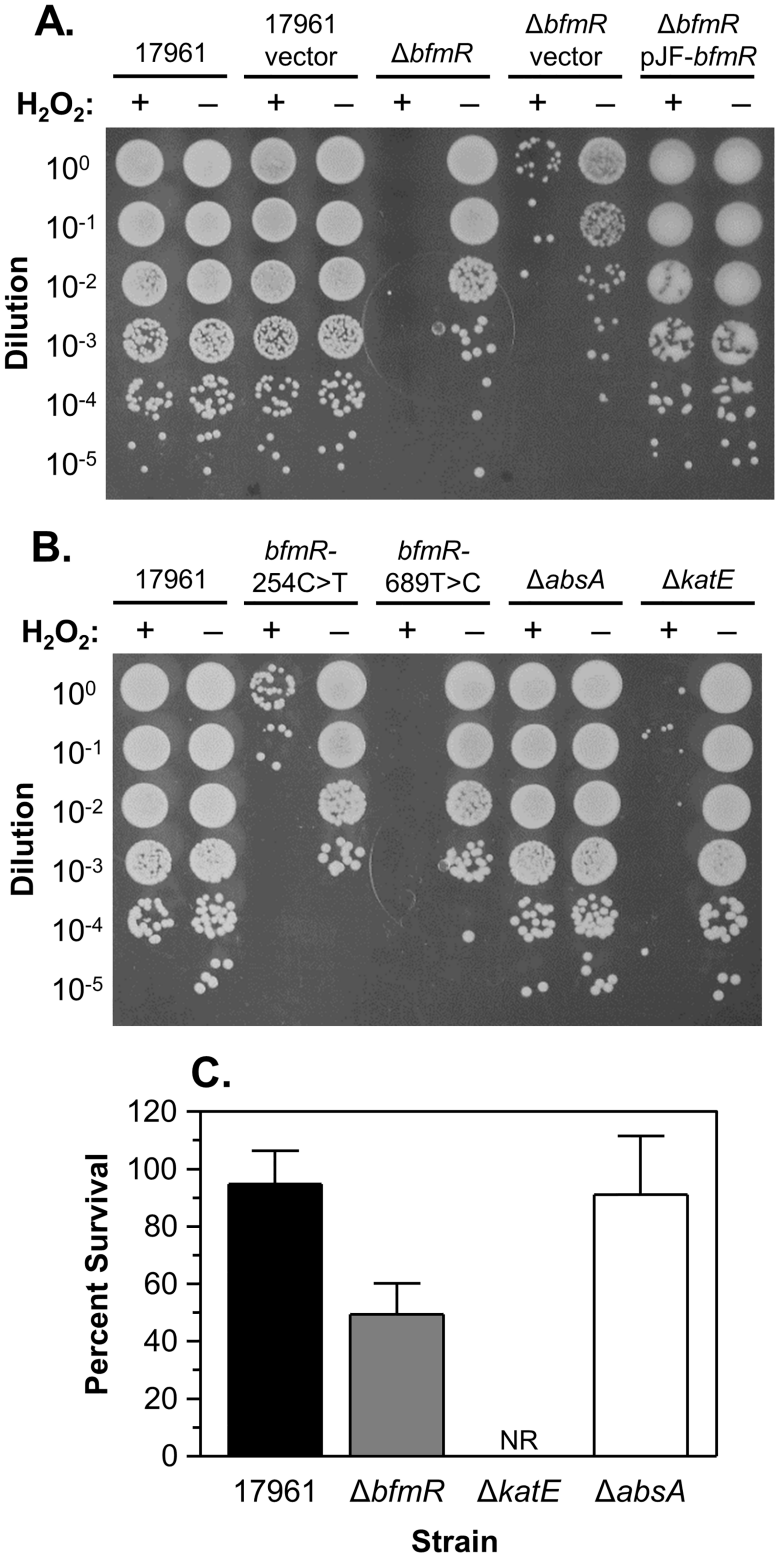
Sensitivity of *A*. *baumannii* cells to hydrogen peroxide. (A) and (B) Dried bacterial samples were challenged with H_2_O_2_. The indicated *A*. *baumannii* strains were grown overnight in LB medium. Samples of washed cells were dried, and then treated with either water or 1.5% H_2_O_2_, as indicated, for 45 s. Each sample was then diluted and spotted onto the surface of an LB plate. These data are representative of at least two independent experiments. (C) Cells in liquid culture were challenged with H_2_O_2_. *A*. *baumannii* strains were grown overnight, and then diluted into fresh LB medium and challenged with 10 mM H_2_O_2_ for 30 min at 37°C. The designation “NR” indicates that no viable cells were recovered after the H_2_O_2_ challenge. The data presented are the mean ± SD from at least four independent experiments.

First, we analyzed the resistance of both dried and broth-cultured cells to hydrogen peroxide treatment. Dried cells of strain 17961 were resistant to H_2_O_2_ treatment, with samples showing only a slight decrease in viability after challenge with 1.5% H_2_O_2_ (0.49 M) for 45 sec (Fig 5A). In contrast, samples of the Δ*bfmR*, *bfmR*-254C>T, and *bfmR*-689T>C mutants had large decreases in survival after H_2_O_2_ treatment (Fig 5A and 5B). Resistance to hydrogen peroxide was restored in the Δ*bfmR* mutant carrying an intact copy of *bfmR* on a plasmid, but not with the vector plasmid alone (Fig 5A). Similarly, cells from the Δ*bfmR* mutant had a reduced ability to survive H_2_O_2_ treatment in broth culture as well. After 30 min in the presence of 10 mM H_2_O_2_, the Δ*bfmR* mutant had approximately a 50% decrease in survival compared with only a 5% decrease in the survival of the wild-type strain (Fig 5C). These results showed that BfmR is important for hydrogen peroxide resistance in *A*. *baumannii* strain 17961. Additionally, we tested the susceptibility of the Δ*absA* and Δ*katE* mutant strains to H_2_O_2_. The Δ*absA* mutant strain had a similar level of resistance to H_2_O_2_ treatment as the wild-type strain, and as expected the Δ*katE* mutant was highly sensitive to H_2_O_2_ both when dried and in liquid culture (Fig 5B and 5C, respectively). This demonstrated that KatE is critical for the hydrogen peroxide resistance of stationary-phase cells in *A*. *baumannii* strain 17961, and also suggests that the increased susceptibility of *bfmR* mutants to H_2_O_2_ treatment is due to decreased levels of KatE in these strains.

**Fig 6:**
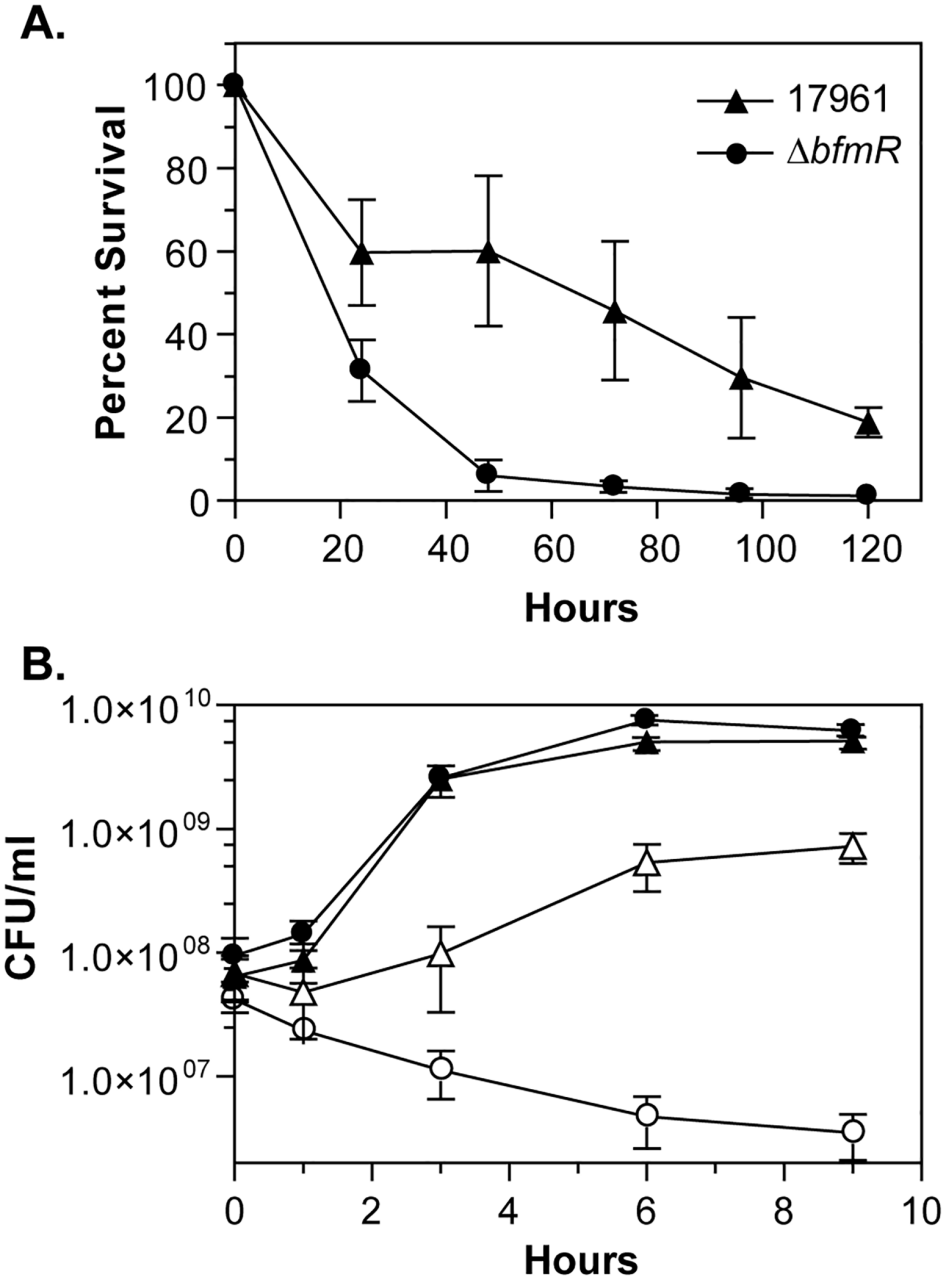
BfmR is important for survival during nutrient limitation and conditions of high osmolarity. (A) The survival of the wild-type strain ATCC 17961 (triangles) and the *bfmR* mutant 17961-Δ*bfmR* (circles) incubated at 37°C in PBS was assessed by counting the number of CFU recovered at each time point. The data presented represent the mean ± SD from three separate experiments. (B) The growth of the wild-type (triangles) and the Δ*bfmR* mutant strains (circles) were monitored at 37°C in the absence (closed symbols) or presence (open symbols) of 0.6 M NaCl. The data presented are the mean ± SD from at least four independent experiments.

Next, we tested the ability of the wild-type and Δ*bfmR* mutant strains to survive during an extended period of nutrient deprivation. For each strain, cells from overnight cultures grown in LB medium were washed and incubated in phosphate-buffered saline for five days. During the first day, cultures of strain 17961 had an approximately 40% decrease in the number of viable cells, followed by a continued slow decline in viability over the next four days (Fig 6A). However, the Δ*bfmR* mutant strain had an approximately 94% decrease in survival during the first 48 hours of nutrient starvation (Fig 6A). These data showed that BfmR is necessary for optimal survival in the absence of nutrients. We also analyzed the growth of the Δ*bfmR* mutant strain under conditions of increased osmolarity. The wild-type and Δ*bfmR* mutant strains both grew equally well in LB medium without sodium chloride (Fig 6B). When strain 17961 was shifted from medium without NaCl to medium containing 0.6 M NaCl there was initially a small decrease in viability, but the cells were able to adapt to the high salt conditions and resume growth, although they did not grow to as high of a density as in media without NaCl (Fig 6B). Unlike the wild-type strain, the Δ*bfmR* mutant was not able to resume growth after being placed in media containing 0.6 M NaCl, and instead these cultures had a slow decrease in cell viability throughout the course of the experiment (Fig 6B), indicating that BfmR is required for cells to adapt to high salt conditions. Together, these results showed that BfmR is important for *A*. *baumannii* to survive under multiple stressful conditions.

**Fig 7:**
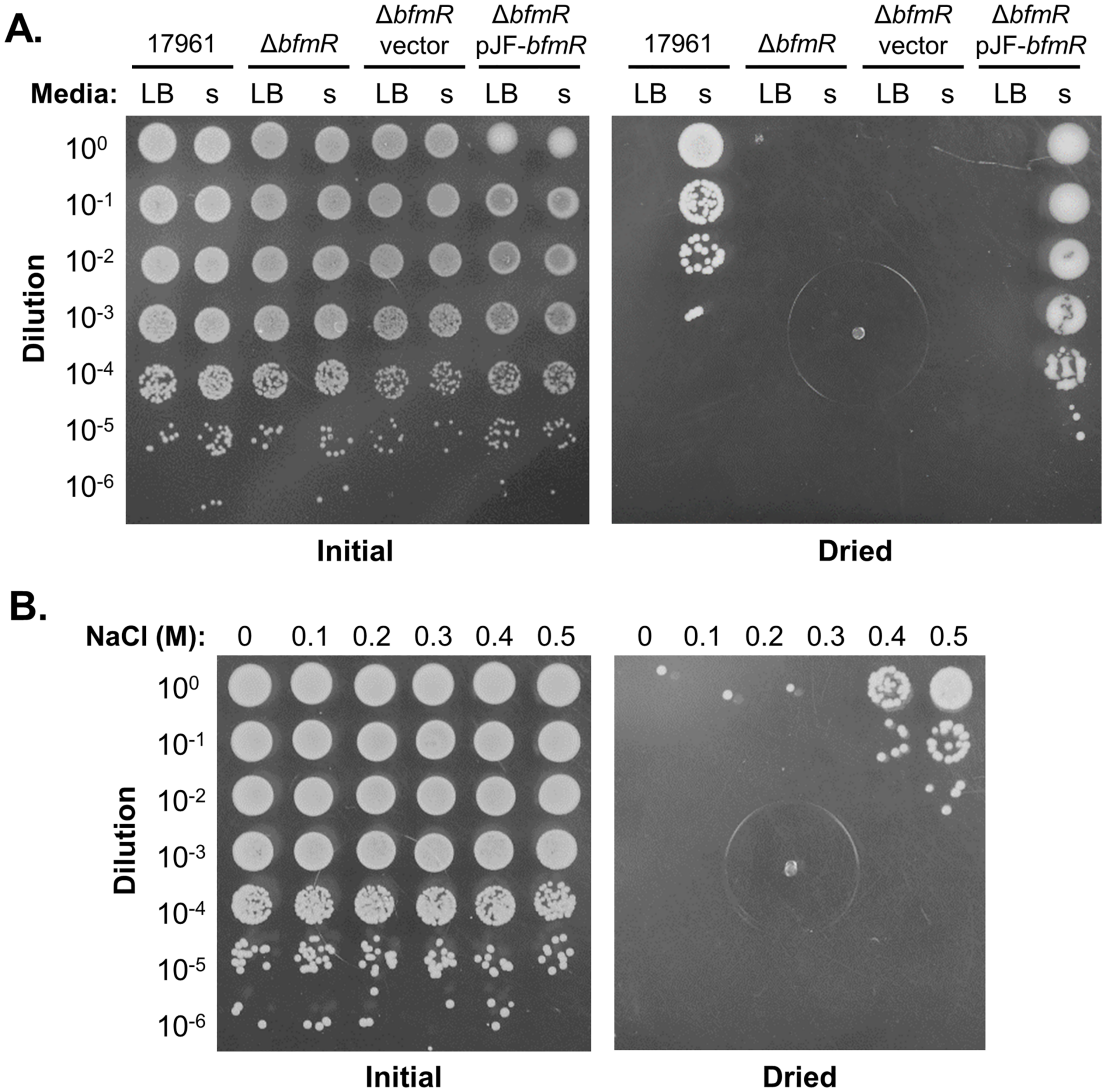
Nutrient starvation and increased osmolarity can stimulate cross-protection against desiccation. (A) *A*. *baumannii* cells in the exponential phase of growth were diluted 1:5 either into fresh growth medium (LB) or phosphate-buffered saline (s). After one hour of incubation at 37°C, samples of cells were taken from each culture, washed with water, and dried for one day. “Vector” refers to the cloning vector pWH1266, and pJF-*bfmR* carries an intact copy of *bfmR*. (B) *A*. *baumannii* strain ATCC 17961 was grown in LB without NaCl, and then diluted 1:5 into LB supplemented with the indicated concentrations of NaCl. After one hour of incubation at 37°C, samples of cells were taken from each culture, washed with water, and dried. For both experiments, samples from the initial inoculum and after one day of drying were diluted and spotted onto the surface of an LB plate. The data presented are representative of at least two independent experiments.

Finally, we tested whether responses to other stresses can promote cross-protection against desiccation in *A*. *baumannii*. To do this, we challenged cells during the logarithmic phase of growth, when they are more sensitive to desiccation (Fig 1B), with either nutrient starvation or increased osmolarity, and then compared the ability of stressed and unstressed cells to survive drying. For starvation experiments, *A*. *baumannii* strains were grown in phosphate-buffered LB for 2.5 hours, and then samples of cultures were diluted 1 to 5 either into fresh medium or phosphate-buffered saline, which was at the same temperature, pH, and NaCl concentration as the original growth medium. One hour after this transfer, cells from each condition were tested for desiccation tolerance. Cells of strain 17961 that were sub-cultured in LB were very sensitive to drying, with few to no viable cells recovered after being dried for one day, but cells that were starved by sub-culture in buffer were much more resilient to desiccation (Fig 7A). We did not observe this effect in the Δ*bfmR* mutant strain, which was still highly sensitive to desiccation after starvation (Fig 7A). As expected, the Δ*bfmR* mutant carrying an intact copy of *bfmR* on a plasmid was protected from drying after starvation (Fig 7A). To test the effects of osmolarity, strain 17961 was initially grown in LB without NaCl for 2.5 hours, and then cells were diluted into pre-warmed LB medium containing varying amounts of NaCl. After one hour of incubation, cells from cultures containing 0.4–0.5 M NaCl had an increased ability to survive drying compared with cultures containing lower amounts of NaCl (Fig 5B). We were unable to clearly assess whether BfmR was necessary for this effect, since the viability of the Δ*bfmR* mutant greatly decreased after one hour of incubation with 0.5M NaCl. Nevertheless, these experiments indicated that responses to both starvation and increased osmolarity can provide cross-protection against drying in *A*. *baumannii*, and that BfmR is required for this effect in response to general nutrient starvation.

## Discussion

Desiccation tolerance is considered to be one of the abilities that has allowed *A*. *baumannii* to become a successful opportunistic pathogen in the nosocomial environment, but the molecular mechanisms that contribute to this trait are poorly understood. While many *A*. *baumannii* strains have been reported to survive drying for 20 days or longer [20], clinical isolates of *A*. *baumannii* can vary in their ability to survive desiccation similar to the group of strains analyzed in this study [21, 35]. Several mechanisms have been described to account for phenotypic variations in *A*. *baumannii* strains that occur during both culture in the laboratory and during infection, including plasmid loss, mobilization of genetic elements, and phase change, which helps to partly explain this organism’s naturally occurring variability [27, 28, 36]. For profoundly drying-resistant strains we found that a much larger proportion of the bacterial population survived drying for an extended period of time, suggesting that strains which exhibit this phenotype could have more opportunities for survival and spread in the hospital setting. However, we also observed that this phenotype could be lost after growth in liquid medium. A previous study noted that *A*. *baumannii* strains isolated from dry sources tended to survive desiccation better than strains isolated from wet sources [35], and together these findings suggest that desiccation can be an important selective pressure in the environment.

Through an analysis of strains that lost the ability to survive drying during laboratory culture we were able to identify BfmR as a regulator of the desiccation resistance phenotype. In *A*. *baumannii*, BfmR is part of a two-component regulatory system with the sensor histidine kinase BfmS, which appears to inactivate BfmR through phosphorylation [30]. Originally identified as having a role in controlling biofilm formation, *bfmR* and *bfmS* have subsequently been linked to several phenotypes, including formation of pili, motility, complement resistance, antibiotic susceptibility, and virulence [29, 30, 37–41]. BfmR appears to be similar to other response regulators in the OmpR/PhoB family, and based on structural data [39, 42] one of the naturally-occurring mutations that we found (in the “switch” threonine, Thr85) likely affected BfmR function by disrupting conformational changes in the protein that occur in response to phosphorylation. The effects of the other mutation we observed, in Leu230, are unclear, but both mutations appear to have altered BfmR’s regulatory activity, leading to increased sensitivity to desiccation (Fig 2). There is currently interest in developing compounds to target BfmR in order to treat *A*. *baumannii* infections [39, 42], and our findings provide evidence that this could be a potential strategy to control *A*. *baumannii* in the healthcare environment as well.

BfmR and BfmS were previously shown to influence phenotypes which could potentially affect drying survival. BfmR was originally identified as a regulator of biofilm formation [29], and another study found that *A*. *baumannii* strains that formed robust biofilms were more desiccation tolerant than strains that could not form biofilms [25]. However, in our studies broth-grown cells were washed thoroughly and then samples were dried rapidly, typically in approximately 45 minutes. This procedure provided little time for the cells to respond to the change in water availability and form a complex biofilm matrix. Therefore we believe that a difference in biofilm formation is an unlikely explanation for the significant drying survival defect of the *bfmR* mutant strains in our experiments (Fig 2), although biofilm formation likely plays a role in the persistence of *A*. *baumannii* in the nosocomial environment. Capsule production is another phenotype influenced by BfmRS that could contribute to desiccation tolerance, since exopolysaccharides are known to aid a variety of bacterial species in drying survival [11]. Strains with mutations that either delete or inactivate *bfmS* have increased capsular polysaccharide production, but this does not occur in *bfmRS* mutant strains, implying a role for BfmR in regulating capsule synthesis [30, 43]. However, another study found that inactivation of *bfmR* alone in two different *A*. *baumannii* strains did not have any effect on capsular polysaccharide production [39]. From these findings it appears that BfmR negatively regulates capsule production in response to phosphorylation by BfmS, but the exact role of BfmR in controlling capsule expression in wild-type strains is still somewhat unclear. While extracellular polysaccharides could certainly help protect *A*. *baumannii* cells during desiccation, the capsule layer surrounding laboratory-grown *A*. *baumannii* cells appears to be relatively thin, and a capsule is produced by both the extremely desiccation-tolerant strain ATCC 17961, and the more desiccation-sensitive strains ATCC 17978 and ATCC 19606 [43]. Based on this, we believe that differences in capsule production are not likely to be the only cause of the large differences in drying survival that we observed. Instead, we hypothesize that these differences are more likely due to the fact that BfmR appears to coordinate multiple stress responses.

Our decision to investigate the role of BfmR in additional stress responses was in response to our discovery that BfmR controlled the expression of two potential stress-related proteins, KatE and AbsA. Both of these proteins were maximally produced during the stationary phase of growth (Fig 3B), which coincided with the time of greatest desiccation tolerance (Fig 1B). Additionally, a recently reported transcriptome analyses of *bfmR* and *bfmS* mutant strains found that the BfmRS system controls a post-exponential growth phase-like pattern of gene expression in the moderately desiccation-resistant strain ATCC 17978 [30]. Several other bacterial species exhibit enhanced desiccation tolerance during stationary phase, and in both *Escherichia coli* and *Pseudomonas fluorescens* the general stress response regulator RpoS is necessary for maximal survival when dried [19, 44–46]. A role for general stress responses in desiccation tolerance makes sense given that drying can adversely affect many different cellular components [14]. General stress responses can provide protection against oxidative stress, starvation, and increased osmolarity [34], and we found that BfmR was important for defense against these stresses as well (Figs 5 and 6). Desiccation itself can also stimulate responses that help protect bacterial cells from both drying and other stresses [47], and *A*. *baumannii* was found to produce higher levels of molecular chaperones and proteins involved in protection from oxidative stress and antibiotic resistance after exposure to dry conditions [26], indicating that a broad range of responses is likely important for *A*. *baumannii* to survive and recover from drying. We additionally found that BfmR was required for starvation-induced cross-protection against drying, and that increased osmolarity can also trigger cross-protection against drying (Fig 7), supporting the notion that decreased desiccation tolerance in the *bfmR* mutant strains was due to a defect in the ability to mount an effective stress response. These results are intriguing since *A*. *baumannii* does not appear to encode an RpoS homolog, and together with the transcriptome data of Geisinger *et al*. [30], our findings imply that general stress responses in *A*. *baumannii* are coordinated by an alternative mechanism that includes the BfmRS two-component signaling system.

While we did not identify all of the BfmR-controlled factors that contribute to desiccation tolerance, we did find that the KatE catalase supports the long-term survival of *A*. *baumannii* cells when dried (Fig 4), which is a novel function for this protein. KatE was previously shown to be important for H_2_O_2_ degradation in *A*. *baumannii* and *A*. *nosocomialis* [31], and we found that KatE was necessary to protect dried cells from H_2_O_2_ treatment (Fig 5). KatE is also required for maximum long-term survival of *E*. *coli* cells in broth culture [33], and a proteomics analysis of *A*. *baumannii* cells under dry conditions found increased levels of KatE during desiccation [26], providing further support for KatE’s role in long-term drying survival. Other genes that are important for defense against oxidative stress, including *sodC*, *ahp*, and *acnA*, were found to be controlled by BfmRS according to transcriptome analyses [30], which also identified some genes that help to explain the susceptibility of the Δ*bfmR* mutant to other stresses as well. In particular, genes involved in the production of compatible solutes, including genes for the synthesis of trehalose and genes for the uptake and accumulation of glycine betaine, were up-regulated by BfmR [30]. Synthesis of compatible solutes is a strategy used by many organisms to protect against both osmotic stress and desiccation, and *A*. *baumannii* produces trehalose, mannitol, and glutamate in response to increased osmolarity caused by challenge with NaCl [48]. Decreased production of compatible solutes could be a potential reason for the inability of the Δ*bfmR* mutant to adapt to high salt condtions (Fig 6B). However, the specific contribution of these compounds to *A*. *baumannii* desiccation tolerance have not yet been defined.

Our current findings are especially interesting based on the potential link between environmental persistence and virulence in *A*. *baumannii*. Stress-related genes, including genes involved in DNA repair, osmotolerance, and nutrient acquisition, were identified in a screen for survival in *Galleria mellonella* larvae, an insect model of infection [41]. The *bfmS* gene was also identified in this screen [41], and both *bfmR* and *bfmS* have been shown to be important for virulence in mammalian infection models [40, 49]. Based on these studies and our current results, we expect that the BfmRS system directs stress responses that are essential for survival in the clinical environment and during the infectious process. However, other regulatory systems likely play a role in these responses as well. The regulators GigA and GigB were also identified as essential for growth in *Galleria*, and are important for resistance to heat and antibiotic-induced stress [50]. Therefore the coordination of *A*. *baumannii* stress responses may involve cross-talk between multiple regulatory systems. Overall, our results show that in addition to its established roles in virulence and biofilm formation, BfmR regulates processes that are important for surviving desiccation, which is an ability that allows *A*. *baumannii* to survive on surfaces in the hospital environment where it can come in contact with the individuals who are most susceptible to infection.

## Materials and methods

### Bacterial strains and growth conditions

The *A*. *baumannii* strains used in this study are listed in Table 2. Stocks of each strain containing 15% glycerol (v/v) were stored at -80°C, and bacteria were freshly plated to begin each experiment. For all experiments bacteria were cultured in lysogeny broth (LB; Lennox formulation), and cultures were incubated at 37°C with shaking at 260–280 rpm. When necessary to maintain plasmids cultures were supplemented with 5 μg/ml tetracycline.

**Table 2:** *A*. *baumannii* strains and plasmids used in this study.

Strain or Plasmid	Description	Reference or source
ATCC 19606	Clinical isolate from urine	ATCC
ATCC 17978	Clinical isolate from spinal meningitis	ATCC
ATCC 17904	Clinical isolate from urine	ATCC
ATCC 17961	Clinical isolate from blood	ATCC
17961-02E8	Laboratory-adapted isolate derived from ATCC 17961	This study
17961-01A8	Laboratory-adapted isolate derived from ATCC 17961	This study
17961-01E3	Laboratory-adapted isolate derived from ATCC 17961	This study
17961-01F2	Laboratory-adapted isolate derived from ATCC 17961	This study
17961-01G4	Laboratory-adapted isolate derived from ATCC 17961	This study
17961-01G8	Laboratory-adapted isolate derived from ATCC 17961	This study
17961-01H12	Laboratory-adapted isolate derived from ATCC 17961	This study
17961-Δ*bfmR*	*bfmR* deletion mutant derived from strain ATCC 17961	This study
17961-*bfmR*-254C>T	ATCC 17961 derivative with a C-to-T substitution in *bfmR* at bp 254	This study
17961-*bfmR*-689T>C	ATCC 17961 derivative with a T-to-C substitution in *bfmR* at bp 689	This study
17961-Δ*absA*	*absA* deletion mutant derived from strain ATCC 17961	This study
17961-Δ*katE*	*katE* deletion mutant derived from strain ATCC 17961	This study
A118	Clinical isolate from blood	[51]
AB09-002	Military isolate from wound culture	G. Plano, Univ. of Miami
AB09-003	Military isolate from cerebrospinal fluid	G. Plano, Univ. of Miami
AB5075	Military isolate from osteomyelitis	[52]
Plasmids		
pEX18Ap	Suicide vector	[53]
pBfmR-suc	*bfmR* region on pEX18Ap	This study
pΔ*bfmR*-suc	Suicide plasmid carrying *bfmR* deletion	This study
pBfmR-254C>T-suc	Suicide plasmid carrying *bfmR* with point mutation	This study
pBfmR-689T>C-suc	Suicide plasmid carrying *bfmR* with point mutation	This study
pΔ*katE*-suc	Suicide plasmid carrying *katE* deletion	This study
pΔ*absA*-suc	Suicide plasmid carrying *absA* deletion	This study
pWH1266	*Escherichia coli-A*. *baumannii* shuttle plasmid	[54]
pJF-*bfmR*	*bfmR* cloned into pWH1266	This study

### Desiccation tolerance assays

Cells were harvested from 1 ml samples of cultures by centrifugation, and then were washed twice with 1 ml of sterile deionized water (dH_2_O). Water was used to avoid additional osmotic stress that could occur from the concentration of salts or buffers during evaporation of the suspending solution. Cell suspensions in dH_2_O were adjusted to an optical density at 600 nm (OD_600_) of 1.0, and then 5 μl droplets of each adjusted suspension were deposited onto a plastic (polystyrene) surface with a micropipette. These samples were left uncovered in a biosafety cabinet at room temperature (21–25°C) until visibly dry, approximately 30 min to 1 h. For experiments to determine the maximum survival time of dried *A*. *baumannii* cells, dried samples were incubated in a dark drawer at ambient temperature and humidity (19–23°C, 25–61% RH), and conditions of temperature and humidity were monitored every other day with a Fisherbrand Traceable relative humidity/temperature meter (Thermo Fisher Scientific). For all other experiments, dried samples were incubated in the dark in a sealed chamber at 24–26°C and at an equilibrium relative humidity 79–81%, which was achieved by storing samples above a saturated solution of sodium chloride within the chamber. Conditions of temperature and humidity within the chamber were monitored with a Fisherbrand Traceable relative humidity/temperature meter (Thermo Fisher Scientific) several times a week. To determine the initial number of cells present, 5 μl samples of each washed and OD_600_-adjusted bacterial suspension were diluted to a volume of 20 μl with saline solution (0.9% sodium chloride), and were then diluted serially and plated onto LB agar. To determine viability after drying, 20 μl of saline was placed onto each dried sample, and the samples were incubated at room temperature for 5 min to rehydrate. The bacteria were suspended by pipetting and gentle scraping to remove the cells from the plastic surface, and the suspended cells were diluted serially in saline and plated onto LB agar. For qualitative assessments, 10 μl of each dilution was spotted onto the surface of the agar, and the plate was incubated at 37°C overnight. For quantitative assessments, dilutions were spread onto agar and the number of viable cells was determined by CFU counts. Percent survival was calculated by comparing the number of CFU recovered from each dried sample to the initial number of CFU present in 5 μl of washed and OD_600_-adjusted cells suspensions prior to drying. At least two dried samples per time-point were suspended and plated for quantitative assays. To determine the maximum survival time of desiccated samples, one dried sample was suspended every other day, and the entire volume was plated onto LB agar. Strains were considered no longer viable if no bacteria were recovered from two consecutive platings.

### Screen for spontaneous mutants and genomic analysis

To isolate spontaneous desiccation-sensitive mutants, freshly plated bacteria were used to inoculate 10 ml of LB medium, and cultures were incubated at 37°C. Approximately every 24 h, 0.2 ml of each culture was used to inoculate 10 ml of LB, and this was continued for a total of five to seven passes in liquid medium. Samples from these cultures were diluted and plated to obtain isolated colonies, and then bacteria from single colonies was used to inoculate 150 μl of LB in 96-well plates. Cultures in 96-well plates were incubated at 37°C with shaking for 6 h. Next, 20 μl from each well was diluted into 100 μl dH_2_O, and 5 μl of these diluted samples was dried in the wells of 96-well round-bottom plates. The remainder of each 96-well plate culture was supplemented with glycerol to achieve a final concentration of 12%, and stored at -80°C. Dried samples were stored in the dark at ambient temperature and humidity for 20 days. After this, 120 μl of LB medium was placed onto each dried sample, and they were incubated at 37°C overnight. Wells were scored visually for growth to identify clones that had lost the ability to survive desiccation. Potentially drying-sensitive clones were then screened using our standard desiccation tolerance assay described above.

To determine the mutations present in desiccation-sensitive isolates that were identified through screening, chromosomal DNA from the parent strain ATCC 17961 and its derivatives was analyzed by next-generation DNA sequencing. Libraries for sequencing were prepared with a TruSeq DNA library prep kit (Illumina), and samples were sequenced using the Illumina MiSeq system by the Genomic Sciences Laboratory at North Carolina State University, Raleigh, NC. Sequencing reads were filtered and trimmed for quality, and adapter sequences were removed using Trimmomatic v0.32 [55]. DNA sequences were analyzed using resources available on the PATRIC website [56]. A draft genome assembly for strain ATCC 17961 was generated using the PATRIC genome assembly service with the “miseq” assembly strategy. The assembled contigs were reordered using the Mauve aligner [57], with the AB5075 genome sequence as a reference. The reordered genome sequence was then annotated with the PATRIC genome annotation service to complete the draft genome. Genetic variations in the drying-sensitive derivatives of strain 17961 were identified through the PATRIC variation analysis service by aligning Illumina sequencing reads to the 17961 genome using BWA-MEM [58], followed by variant calling using either FreeBayes [59] or SAMtools [60]. Variation analyses were performed using the default settings on the PATRIC website. Genetic variations that were identified in either repetitive or duplicated regions of the genome were ignored. Other potential variations were confirmed by PCR amplification and sequencing of the genomic region of interest.

### Construction of plasmids and mutant strains

To construct plasmids for generating strains with mutations in *bfmR*, an approximately 2.5 kb DNA fragment containing the *bfmR* gene was amplified by PCR using chromosomal DNA from strain ATCC 17961 as a template. The oligonucleotide primers for this reaction were designed to add a PstI site to each end of the fragment (S1 Table). Both the DNA fragment and the vector plasmid pEX18Ap were digested with PstI, and the two were ligated together to produce pBfmR-suc. Next, plasmid pBfmR-suc acted as a template for PCR reactions using oligonucleotide primers with 5’-phosphate groups (S1 Table). The DNA fragments generated from these reactions were circularized by ligation to produce plasmids pΔ*bfmR*-suc, pBfmR-254C>T-suc, and pBfmR-689T>C-suc. Plasmid pΔ*bfmR*-suc carries a mutated allele of *bfmR* with an in-frame deletion that removes the DNA sequence coding for amino acids 12 to 237 of BfmR (95% of the protein sequence). Plasmids pBfmR-254C>T-suc and pBfmR-689T>C-suc carry mutant *bfmR* alleles with single nucleotide substitutions at either bp 254 (C to T) or bp 689 (T to C), respectively, of the *bfmR* coding sequence. DNA fragments carrying in-frame deletions in either *absA* or *katE* were generated by PCR as described previously [61]. These fragments were designed to remove the DNA sequence coding for amino acids 26 to 388 of AbsA (88% of the protein sequence) or 41 to 680 of KatE (90% of the protein sequence). The fragments were digested with the appropriate restriction enzyme (PstI for Δ*absA* or EcoRI for Δ*katE*), and then were ligated with pEX18Ap, which had been digested with the same enzyme, to produce plasmids pΔ*absA*-suc and pΔ*katE*-suc.

To construct a plasmid carrying the wild-type *bfmR* gene for genetic complementation, a DNA fragment that began 470 bp upstream from the *bfmR* translational start site and ended 17 bp downstream from the *bfmR* stop codon was amplified by PCR. The oligonucleotide primers for this amplification were designed to contain an EcoRI site at the upstream end of the fragment, and a PstI site at the downstream end (S1 Table). The DNA fragment was digested with these enzymes, and then ligated with pWH1266, which had been digested with the same enzymes, to produce plasmid pJF-*bfmR*.

Mutant strains of *A*. *baumannii* were generated using the procedures described by Hoang *et al*. [53], with some modifications. Plasmids carrying mutant alleles were transferred into *A*. *baumannii* via conjugation with *Escherichia coli* strain SM10. *A*. *baumannii* cells that integrated the plasmid sequence onto the chromosome were selected by plating onto LB supplemented with 150 μg/ml carbenicillin and 5 μg/ml chloramphenicol. Next, mutants were selected by plating carbenicillin-resistant isolates onto LB supplemented with 10% sucrose to resolve plasmid sequences from the chromosome. Potential mutant strains were confirmed by PCR amplification and DNA sequencing of the chromosomal region of interest. For complementation, replicative plasmids were transferred into *A*. *baumannii* strains by electroporation.

### Protein analysis and identification

Cells were harvested from cultures by centrifugation and stored at -80°C until analysis. To extract soluble proteins, each cell pellet was suspended in an appropriate volume (10 μl per mg wet pellet weight) of BugBuster protein extraction reagent (EMD Millipore) supplemented with 2 mg/ml lysozyme and Benzonase nuclease (EMD Millipore), as directed by the manufacturer. Samples were incubated with gentle mixing for 20 min at room temperature, and then were centrifuged at 20,000 × g for 20 min, 4°C. The supernatant (soluble fraction) was collected, and solubilization buffer (50 mM Tris pH 6.8, 1 mM EDTA, 100 mM NaCl, 0.5% SDS) was added to each pellet at a volume equal to the amount of BugBuster reagent used for cell lysis. Pellets were incubated at 55°C for 15 min, and then homogenized to produce the insoluble fraction for analysis. Soluble and insoluble fractions were analyzed by SDS-PAGE on a 12% polyacrylamide gel, and gels were visualized by staining with Coomassie-R250. To identify proteins, individual bands were excised from the gels, and proteins within these gel slices were identified by mass spectrometry (performed by the Proteomics Core Facility at the University of California, Davis).

### Hydrogen peroxide sensitivity assays

To assess the sensitivity of dried *A*. *baumannii* cells to hydrogen peroxide, cells were harvested from overnight cultures by centrifugation, and then were washed twice with sterile dH_2_O. Cell suspensions in dH_2_O were adjusted to an OD_600_ of 1.5, and then 5 μl samples were spotted onto a plastic surface. These samples were incubated uncovered in a biosafety cabinet until visibly dry, 1–2 h. Dried samples were treated with 10 μl of either dH_2_O or 1.5% H_2_O_2_ for 45 sec. Next, 10 μl of 1.5% sodium thiosulfate pentahydrate (STS) was added to each sample to neutralize H_2_O_2_, and bacterial cells were gently suspended by pipetting. Suspended samples were mixed with 180 μl of 1.5% STS, and then suspensions were diluted serially in 1.5% STS. Controls were also performed to ensure that STS did not affect bacterial viability. To assess survival, 10 μl of each dilution was spotted onto the surface of a LB agar plate, and the plate was incubated at 37°C overnight.

To measure the survival of *A*. *baumannii* cells after hydrogen peroxide challenge in broth culture, cells from overnight cultures were used to inoculate fresh LB medium, which was warmed to 37°C, to a density of approximately 1 × 10^8^ CFU/ml. Samples of inoculated medium were supplemented with 10 mM H_2_O_2_, and were incubated at 37°C for 30 min. Samples taken both prior to and after H_2_O_2_ treatment were diluted serially in 1% STS and plated onto LB medium. The number of viable cells was determined by CFU counts, and percent survival was calculated by comparing the number of CFU recovered after treatment with H_2_O_2_ to the number present prior to treatment.

### Nutrient deprivation and osmotolerance assays

For experiments to measure the survival of *A*. *baumannii* strains after extended nutrient starvation, cells from overnight cultures were used to inoculate 10 ml of LB to a density of approximately 6 × 10^6^ CFU/ml. These cultures were incubated at 37°C for 6 h. Then 2 ml samples of each culture were washed twice with 2 ml of sterile phosphate-buffered saline (PBS). Washed cells were suspended in 25 ml PBS at a density of approximately 2.5–3 × 10^8^ CFU/ml, and were incubated with shaking at 37°C. At various time-points samples were taken from the suspensions, diluted serially in PBS, and plated onto LB medium to determine the number of viable cells. Percent survival was calculated by comparing the number of CFU recovered at each time-point to the number of CFU present at T_0_.

To test for tolerance of changes in osmolarity, *A*. *baumannii* cells were grown overnight at 37°C in LB medium without sodium chloride. Cells from overnight cultures were subcultured in 37°C LB medium either without or with 0.6M NaCl at a density of 0.5–1 × 10^8^ CFU/ml. Cultures were incubated at 37°C. At various time-points samples were taken from cultures, diluted serially in 0.5X PBS, and plated onto LB medium to determine CFU/ml.

### Cross-protection assays

Cells from overnight cultures were used to inoculate fresh growth medium to an OD_600_ of 0.05, and cultures were incubated at 37°C for 2.5 h. Then 2.5 ml of this culture was diluted into 10 ml of either fresh growth medium or medium to provide a stress, which had been prewarmed to 37°C. For nutrient starvation, cells were cultured in LB buffered with 10 mM potassium phosphate, pH 7.0, and then diluted into either the same medium or into a buffer containing 10 mM potassium phosphate, pH 7.0, 5 g/l NaCl. To test the effects of osmolarity, cells were grown in LB without NaCl, and then diluted into LB supplemented with NaCl as indicated in the text. After transfer, cultures were incubated at 37°C for 1 h, and then cells were harvested from approximately 1.7 ml of each culture, washed with 1 ml dH_2_O, and suspended in 0.25–0.5 ml dH_2_O. Cell suspensions were adjusted to an OD_600_ of 1.0 with dH_2_O, and were assayed for desiccation tolerance as described above.

### Statistical analysis

Statistical significance was determined using Student’s *t*-test where appropriate, with *P* < 0.05 considered significant. Analyses were performed using Microsoft Excel.

### Accession numbers

Illumina sequencing reads from *A*. *baumannii* strain ATCC 17961, and the drying-sensitive isolates derived from strain ATCC 17961, were deposited in the NCBI Sequence Read Archive under BioProject PRJNA437968, accession numbers SRX3783010, SRX3783011, SRX3783012, SRX3783013, SRX3783014, SRX3783015, SRX3783016, and SRX3783017. The draft genome sequence for strain ATCC 17961 is available on the PATRIC website (http://www.patricbrc.org), Genome ID 470.2202.

## Supporting information

S1 Table Primers used in this study. (PDF)

S1 Fig Desiccation survival phenotypes of various *A*. *baumannii* strains. *A*. *baumannii* strains were grown overnight in LB medium, and then cells were washed with water and samples were dried on polystyrene. Dried cells were incubated at 25°C and 80% RH and, at the indicated times, dried samples were suspended and survival was assessed by CFU counts. For data with error bars, the data represent the mean ± SD from at least three independent experiments. For data without error bars, the data are representative of at least two independent experiments.
(PDF)
