# Supplementary material for: Desiccation tolerance in Acinetobacter baumannii is mediated by the two-component response regulator BfmR
Source: PLoS One. 2018 Oct 11;13(10):e0205638. doi: 10.1371/journal.pone.0205638 (PMC6181384; doi:10.1371/journal.pone.0205638)
Supplement: S1 Table — (PDF) [file pone.0205638.s001.pdf]

Table S1. Primers used in this study.

Primer	Sequence (5'→3')[a, b]
Generation of mutant strains	
bfmR-P1	aaaaaaaactgcagaggaacacgtaatgacaaatcacc
bfmR-P2	ttgtaaaatctgattaaacttcctataaggttg
bfmR-P3	cagaatctttggtaacttttcttcttggct
bfmR-P4	aaaaaaaactgcagttgatcgcgatagataacgtaagc
bfmRC254T InvF	caacattaagattggttgatgatag
bfmRC254T InvR	aTtgcgcgtactgaagatatgg
bfmRT689C InvF	gtaacctttactacgtacag
bfmRT689C InvR	tCgtttgttaaagaaaccaatgg
AbSP-P1	aaaactgcagggcatctaacatagacaacgaattgg
AbSP-P2	cgtggatttgcaagtatggatccttcagatggtcgtggccagaactctc
AbSP-P3	gagagttctggccacgaccatctgaaggatccatacttgcaaatccacg
AbSP-P4	aaaactgcagctttcaacaattgatcgattacctg
katE-P1	aaaagaattcttggctaaatgatacaggtaatcg
katE-P2	gttagacacagttagagatgatgcaattgaagatgagttccagcatgttg
katE-P3	caacatgctggaactcatcttcaattgcatcatctctaactgtgtctaac
katE-P4	aaaagaattcaccatgtctgtatctatgatgacc
Cloning *bfmR* for complementation	
bfmR comp F	ttttttctgcagggaagtttaatcagattttacaatccattgg
bfmR comp R	tttttttgaattcatgccacgatatttgagggataaacac

[a]Underlined sequence denotes a restriction site utilized for cloning.

[b]Nucleotides shown in capital letters are altered compared to the original template sequence.
