# Supplementary material for: Desiccation tolerance in Acinetobacter baumannii is mediated by the two-component response regulator BfmR
Source: PLoS One. 2018 Oct 11;13(10):e0205638. doi: 10.1371/journal.pone.0205638 (PMC6181384; doi:10.1371/journal.pone.0205638)
Supplement: S1 Fig — A. baumannii strains were grown overnight in LB medium, and then cells were washed with water and samples were dried on polystyrene. Dried cells were incubated at 25°C and 80% RH and, at the indicated times, dried samples were suspended and survival was assessed by CFU counts. For data with error bars, the data represent the mean ± SD from at least three independent experiments. For data without error bars, the data are representative of at least two independent experiments. (PDF) [file pone.0205638.s002.pdf]

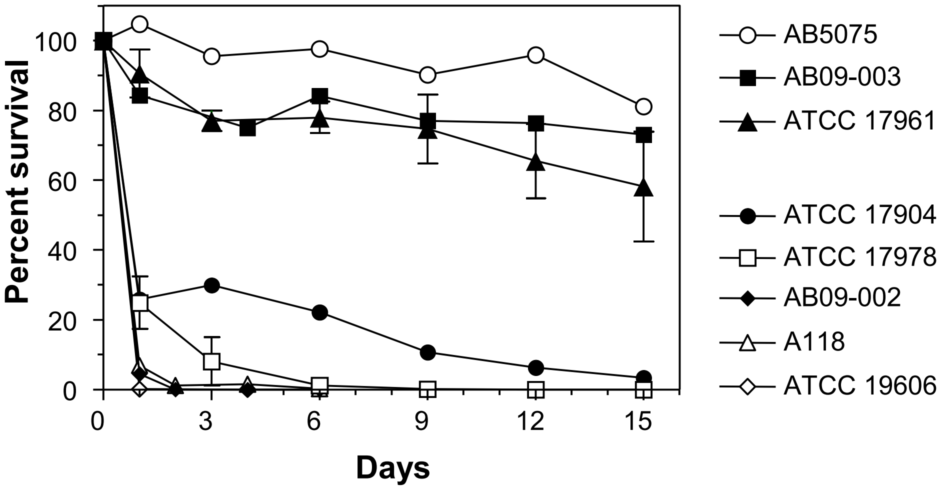

S1 Fig. Desiccation survival phenotypes of various *A. baumannii* strains. *A. baumannii* strains were grown overnight in LB medium, and then cells were washed with water and samples were dried on polystyrene. Dried cells were incubated at 25°C and 80% RH and, at the indicated times, dried samples were suspended and survival was assessed by CFU counts. For data with error bars, the data represent the mean ± SD from at least three independent experiments. For data without error bars, the data are representative of at least two independent experiments.
